# Characteristics and Mediating Effect of Gut Microbiota With Experience of Childhood Maltreatment in Major Depressive Disorder

**DOI:** 10.3389/fnins.2022.926450

**Published:** 2022-06-14

**Authors:** Yanyan Zhang, Ruiyu Zhang, Penghong Liu, Jizhi Wang, Mingxue Gao, Jie Zhang, Jun Yang, Chunxia Yang, Yu Zhang, Ning Sun

**Affiliations:** ^1^Department of Psychiatry, First Hospital of Shanxi Medical University, Taiyuan, China; ^2^Department of Physiology, Shanxi Medical University, Taiyuan, China; ^3^Key Laboratory of Cellular Physiology, Ministry of Education, Shanxi Medical University, Taiyuan, China; ^4^First Clinical Medical College of Shanxi Medical University, Taiyuan, China

**Keywords:** major depressive disorder, childhood maltreatment, gut microbiota, mediating analysis, diversity and inclusion

## Abstract

Gut microbiota and childhood maltreatment are closely related to depressive symptoms. This study aimed to analyze the characteristics of gut microbiota in major depressive disorder (MDD) patients with childhood maltreatment experience and explore the correlation between gut microbiota, childhood maltreatment, and depressive symptoms. A total of 37 healthy controls (HCs) and 53 patients with MDD were enrolled, including 18 MDD patients without childhood maltreatment experience and 35 MDD patients with childhood maltreatment experience. The Hamilton’s Depression Scale (HAMD-24) and Childhood Trauma Questionnaire-Short Form (CTQ-SF) were used to evaluate their depressive symptoms and childhood maltreatment experience, respectively. The composition of gut microbiota was evaluated using 16S rRNA sequencing. Spearman’s correlation analysis was used to evaluate the correlation between different gut microbiota, depressive symptoms and childhood maltreatment. The mediation analysis was used to evaluate the mediating effect of gut microbiota. In the α-diversity analysis, we found that the Simpson index and Pielou’s Evenness index differed significantly between MDD patients without childhood maltreatment experience and HCs. In the β-diversity analysis, principal coordinate analysis (PCoA) showed significant differences between MDD patients without childhood maltreatment experience, MDD patients with childhood maltreatment experience and HCs. Twenty-seven different bacteria were identified through Linear discriminant analysis effect size (LEfSe) analysis at different levels of classification. The analysis of the correlation showed that *Blautia, Bifidobacterium, Bacteroides, Roseburia*, and *Phascolarctobacterium* were significantly correlated with HAMD and CTQ-SF scores. The mediation analysis showed that childhood maltreatment had a significant direct effect on the patients’ depressive symptoms, and *Blautia, Bifidobacterium, Roseburia* had a significant mediating effect. The findings of this study suggested that MDD patients with childhood maltreatment experience had different gut microbiota, which might have a mediating effect on the influence of childhood maltreatment on depressive symptoms.

## Introduction

Major depressive disorder (MDD) is a common mental disorder characterized by significant and persistent low mood, loss of interest and anhedonia. According to recent epidemiological studies, approximately 310 million people worldwide suffered from depression,^1^ (World Health Organization, 2021), and more than 700,000 people die by suicide every year, making it the fourth leading cause of death in the population aged 15–29 years^2^ (Institute of Health Metrics and Evaluation, 2019). MDD seriously affects patients’ physical and mental health, reduces their quality of life, and is a major burden on communities globally (Snyder, 2013; Herbert and Lucassen, 2016).

At present, the pathogenesis of MDD still remains unclear. The role of environmental factors in the pathogenesis of MDD has attracted greater attention from physicians and researchers (Foster et al., 2017; O’Mahony et al., 2017). Several studies have shown that people who have experience of physical abuse (PA) or emotional abuse (EA) are more likely to suffer from depression (Biedermann et al., 2021; Husain et al., 2021), and the severity of symptom is strongly associated with childhood maltreatment (Whitaker et al., 2021; Yang et al., 2021; Rep et al., 2022). According to a study, the effects of childhood maltreatment could last for decades and are strongly associated with the onset of mental disorders at an older age (Macpherson et al., 2021). Therefore, childhood maltreatment should be recognized as a key factor in the diagnosis, treatment, research, prevention and education related to mental illnesses (Teicher et al., 2021).

In recent years, the gut microbiota has come into focus. The human gut microbiota is a dynamic and diverse microbial community composed of symbiotic bacteria, fungi, and viruses. These microorganisms regulate many host functions, including dietary fiber fermentation, pathogen defense, metabolism, and immune maturation. Recently, several studies have shown that patients with MDD have gut microbiota disturbance (Naseribafrouei et al., 2014; Jiang et al., 2015; Zheng et al., 2021), and their depression symptoms are associated with changes in the composition of microbiota composition (Kelly et al., 2016; Chen et al., 2021). Although the results of the above studies are not fully consistent, most of the them showed that, compared with healthy controls (HCs), patients with depression had lower *Firmicutes* and higher *Bacteroidetes* levels, increased levels of pro-inflammatory bacteria and decreased levels of anti-inflammatory bacteria (Liu et al., 2020; Mason et al., 2020). In addition, studies have demonstrated that animals receiving feces transplantation from depressed patients showed depression symptoms, indicating that there might be a causal relationship between gut microbiota and depression symptoms (Zheng et al., 2016; Settanni et al., 2021).

In stress-induced animal models of depression, there were significant differences in the composition of gut microbiota between depressed mice and HCs. However, studies using differential gut microbiota are not entirely consistent. The study of Jianguo et al. (2019) showed that *Blautia* was the only genus enriched in depressed rats, however, the study of Wu et al. (2020) found that six species of bacteria were enriched in depressed mice. The most differentially abundant bacteria in depressed mice, taxa, was found to belong to *Allobaculum* (Wu et al., 2020). A number of studies showed that the diversity and composition of gut microbiota in animal models of depression could be reversed by different interventions (Cao et al., 2020; Cheng et al., 2020; Yan et al., 2020; Yue et al., 2021; Zhang et al., 2021).

There are a few studies showing that early life stress is associated with altered behaviors and gut microbiota in animals (Bailey and Coe, 1999; O’Mahony et al., 2009), while there has been few studies on the association between early childhood maltreatment and gut microbiota characteristics in humans with depressive symptoms. Therefore, we analyzed the characteristics of gut microbiota in MDD patients who had experienced childhood maltreatment and their association with depressive symptoms and Childhood Trauma Questionnaire-Short Form (CTQ-SF) score. In this study, we hypothesized that childhood maltreatment might lead to depressive symptoms by disturbing gut microbiota. To test this hypothesis, we built a mediation model, using childhood maltreatment as the independent variable, the depression symptom score as the dependent variable, and gut microbiota as the intermediary variable. Hopefully, the results may provide guidance for further studies on the relationship between childhood maltreatment and depressive symptoms.

## Materials and Methods

### Participants

A total of 90 subjects (aged 18–50 years), including 53 first-episode drug-naïve MDD patients and 37 HCs, were recruited from the First Hospital of Shanxi Medical University. The MDD patients who had not experienced child maltreatment were assigned into a group marked as CTQ0, and the MDD patients who had experienced child maltreatment were assigned into a group marked as CTQ1. All the patients met the criteria for MDD in the Diagnostic and Statistical Manual of Mental Disorders-Fourth Edition (DSM-IV). Hamilton Depression Scale-24 (HAMD-24) was used to measure the severity of depression, and the HAMD score of all MDD patients was greater than 20. The patients also completed the CTQ-SF (Bernstein et al., 2003) to assess their trauma experience before the age of 16. All HCs were recruited through advertisements in the community and on social media, and those with any mental illnesses were excluded. Subjects who had the following conditions were excluded: (1) hypertension, diabetes mellitus, endocrine diseases, fatty liver, gastrointestinal diseases, severe cardiovascular diseases or neuropsychological diseases; (2) use of antibiotics, probiotics, prebiotics or synbiotics within a month prior to enrollment; (3) pregnancy or breastfeeding.

This study was approved by the Ethics Committee of the First Hospital of Shanxi Medical University. All participants provided written consent after provided with detailed information about the study objectives and procedures.

### Childhood Trauma Questionnaire-Short Form Assessment

Childhood Trauma Questionnaire-Short Form (Jiang et al., 2018) is an internationally recognized tool for the assessment of children’s traumatic experience and is widely used for the screening of a history of childhood maltreatment. CTQ-SF consists of five subscales for the separate assessment of EA, PA, sexual abuse (SA), emotional neglect (EN), and physical neglect (PN), with each item rated from 1 (none) to 5 (most serious); thus, the total score of each subscale ranges from 5 to 25 points. For subscale scores, EA > 12, PA > 9, SA > 7, EN > 14, and PN > 9 indicated a history of childhood maltreatment; otherwise, no experience of childhood maltreatment was indicated.

### Fecal Sample Collection and Gut Microbiota Analysis

After the completion of the HAMD-24 and CTQ-SF assessments, we collected a total of 90 fecal samples. To ensure that the samples were not contaminated, the stool was collected from the middle using plastic cups. The specimens were sent to the laboratory within 15 min and immediately stored in a refrigerator at −80°C until analysis.

Total DNA was extracted from the stool specimens using the Stool Mini Kit (QIAGEN, Hilden, Germany). The purity and integrity of DNA were analyzed using 1% agarose gel electrophoresis and the concentration of DNA was quantified. The total DNA was stored in the refrigerator at −20°C until the next analysis.

In order to amplify the bacterial 16S rRNA gene, we used 338F universal primer (5′-ACTCCTACGGGAGGCAGCAG-3′) and 806R (5′-GGACTACHVGGGTWTCTAAT-3′) to amplify V3-V4 of the bacterial 16S rRNA hypervariable region. Personal Biotechnology, Co., Ltd. Illumina MiSeq platform was used for PCR amplification, sequencing library preparation and pyrosequencing (Shanghai, China).

QIIME 2 (version: 2019.1) was used for analysis, including repeated sequence removal, clustering, and chimerism removal. The sequences with 100% similarity were clustered into amplicon sequence variants (ASVs). Based on the Greengenes Database, the representative sequences were annotated, and the community composition of each specimen was counted at the classification level. The abundance and classification of all ASVs were recorded for each specimen.

### Statistical Analyses

The QIIME 2 (version: 2019.1) and SPSS software (version 23.0) were used for data analyses. The QIIME 2 software was used to calculate α-diversity and β-diversity. The α-diversity analysis included Chao1, Faith’s PD, Good’s coverage, Shannon, Simpson, Pielou’s evenness and Observed species indices, while for the β-diversity analysis, principal coordinate analysis (PCoA) based on bray_curtis was used to reveal the structural differences of bacterial communities between different groups. Linear discriminant analysis effect size (LEfSe) analysis was used to identify marker species between groups. The SPSS software was used to analyze the general demographic information and mediating effects. One-way analysis of variance (ANOVA) was used to compare the age, years of education, and body mass index (BMI) between the three groups, and the χ2 test was used to for the comparison of gender distribution. Correlations between gut microbiota, HAMD-24, and CTQ-SF were calculated using Spearman’s rank-correlation analysis. The mediation effect was analyzed using the Process software. For all analyses, the threshold of statistical significance was set at *P* < 0.05.

## Results

### General Demographic Information

The demographic information and clinical characteristics of the patients with MDD and HCs are presented in Table 1. There were no significant differences between the three groups in age (*P* = 0.888), gender (*P* = 0.367), BMI (*P* = 0.625) and years of education (*P* = 0.714), and the scores of HAMD-24 and CTQ-SF were significantly higher among patients with MDD as compared to HCs (*P* < 0.01).

**TABLE 1:** Clinical and demographic characteristics of the HC, CTQ0, and CTQ1 groups.

Variable	HCs (*n* = 37)	CTQ0 (*n* = 18)	CTQ1 (*n* = 35)	F/χ^2^	*P*-value
Gender (M/F)	18/19	7/11	12/23	2.006	0.367
Age (years)	24.16 ± 4.067	24.62 ± 7.830	24.00 ± 5.620	0.119	0.888
BMI (kg/m2)	21.69 ± 2.108	21.52 ± 2.228	21.18 ± 3.548	0.472	0.625
Years of education	16.59 ± 2.164	16.24 ± 3.270	16.16 ± 3.329	0.338	0.714
HAMD-24	2.16 ± 3.172	24.78 ± 4.066	24.74 ± 5.336	342.571	<0.001
CTQ-SF	26.86 ± 1.554	31.52 ± 3.888	54.86 ± 11.249	220.482	<0.001
EA	5.59 ± 0.781	6.66 ± 1.798	13.02 ± 4.579	92.452	<0.001
PA	5.18 ± 0.386	5.21 ± 0.861	8.69 ± 3.785	34.999	<0.001
SA	5.00 ± 0.000	5.00 ± 0.000	6.02 ± 2.351	7.928	= 0.001
EN	5.68 ± 0.936	8.59 ± 2.585	15.45 ± 4.417	162.263	<0.001
PN	5.41 ± 0.565	6.07 ± 1.361	11.69 ± 4.264	80.518	<0.001

*HCs, healthy controls; BMI, body mass index; HAMD-24, Hamilton’s depression scale-24; CTQ-SF, the childhood trauma questionnaire-short form; EA, emotional abuse; PA, physical abuse; SA, sexual abuse; EN, emotional neglect; PN, physical neglect.*

### α-Diversity and β-Diversity

**FIGURE 1:**
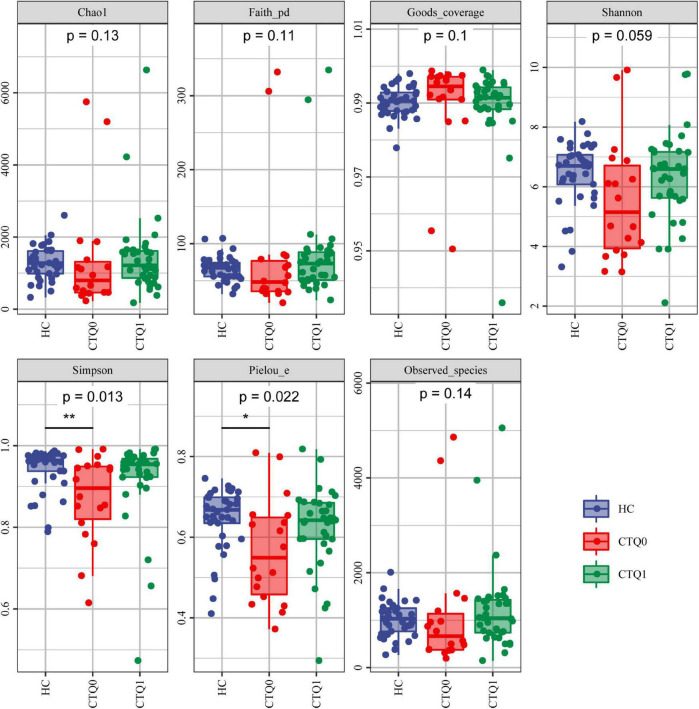
α-diversity of HCs, the CTQ0 group and the CTQ1 groups (marked by blue, red, and green dots and areas, respectively). *P < 0.05, **P < 0.01.

The α-diversity, which reflects species richness, diversity, and evenness in locally homogeneous habitats, was analyzed for the HCs and patients in the CTQ0 and CTQ1 groups. The results showed that the Simpson index and Pielou’s evenness index were higher in HCs than in the CTQ0 group (*P* < 0.05), while no significant differences were found in other indicators between the three groups (Figure 1).

**FIGURE 2:**
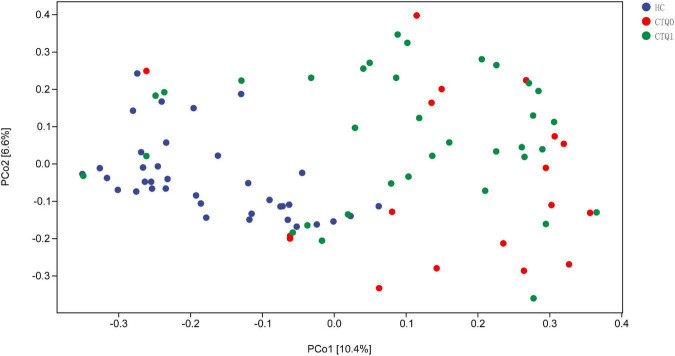
Principal coordinate analysis (PCoA) of β-diversity based on bray_curtis (The HCs, CTQ0 group and CTQ1 group were marked with blue, red, and green dots, respectively).

To explore the differences in the comprehensive microbial phenotypes between HCs, the CTQ0 group and the CTQ1 group, we performed β-diversity analysis. Significant differences were found between the HCs and the CTQ0 group (*P* < 0.001) and between HCs and the CTQ1 group (*P* < 0.001). However, no statistical difference was found between the CTQ0 and CTQ1 groups, as shown in Figure 2.

### Analysis of the Signatures of Gut Microbiota

**FIGURE 3:**
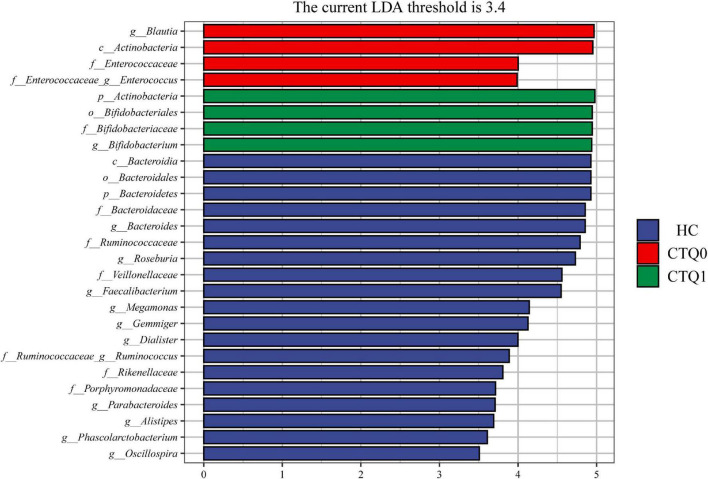
Taxonomic biomarkers found by LefSe analysis in HCs, the CTQ0 group and the CTQ1 group (marked by blue, red, and green color, respectively; the levels of phylum, class, order, family, and genus were indicated with p, c, o, f, and g, respectively, before the name of each microorganism. Only taxa with a *P* < 0.05 and an LDA score over 3.4 is shown).

Linear discriminant analysis effect size (LefSe) analysis was used to identify differences in species and marker species of the three groups. At the family level, the abundance of the *Bifidobacteriaceae* was higher in the CTQ1 group, and the quantity of the *Enterococcaceae* was higher in the CTQ0 group, while the abundance of the *Bacteroidaceae, Ruminococcaceae, Veillonellaceae, Rikenellaceae*, and *Porphyromonadaceae* were higher in HCs group. At the genus level, the abundance of the *Bifidobacterium* was higher in the CTQ1 group, the quantity of the *Blautia* and *Enterococcus* was higher in the CTQ0 group, while the quantity of the *Bacteroides, Roseburia, Faecalibacterium, Megamonas, Gemmiger, Dialister, Ruminococcus, Parabacteroides, Alistipes, Phascolarctobacterium*, and *Oscillospira* was higher in the HCs (Figure 3).

### Relationship Between Gut Microbiota and Clinical Parameters

**FIGURE 4:**
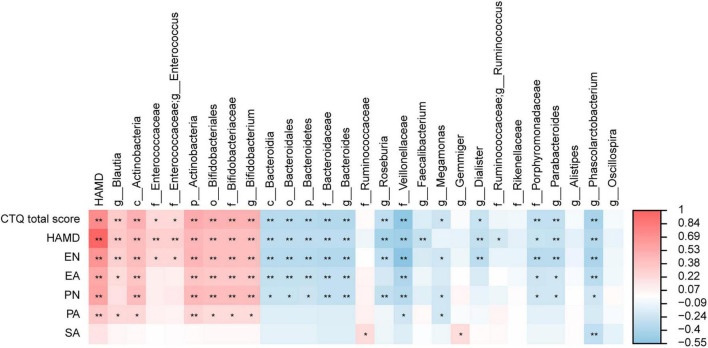
The heatmaps of spearman correlation coefficient matrix between significant different gut microbiota and the score of the HAMD-24 and CTQ-SF. Red indicates positive correlations while blue indicates negative. *P < 0.05, **P < 0.01.

We analyzed the correlation between the different gut microbiota and HAMD-24, CTQ, and five dimensions of childhood maltreatment to identify the marker species. The results showed that HAMD-24 was significantly correlated with CTQ-SF, EN, EA, PN, PA. At the genus level, *Blautia* and *Bifidobacterium* were significantly positively correlated with HAMD-24 and CTQ-SF, *Roseburia, Bacteroides*, and *Phascolarctobacterium* were significantly negatively correlated with HAMD-24 and CTQ-SF (Figure 4). In addition, *Bifidobacterium* was significantly positively correlated with EA and EN, *Phascolarctobacterium* was significantly negatively correlated with EA, SA, and EN, *Blautia* was significantly positively correlated with EN, *Roseburia, Bacteroides*, and *Dialister* were negatively correlated with EN, at the genus level.

### Analysis of Mediating Effect

**FIGURE 5:**
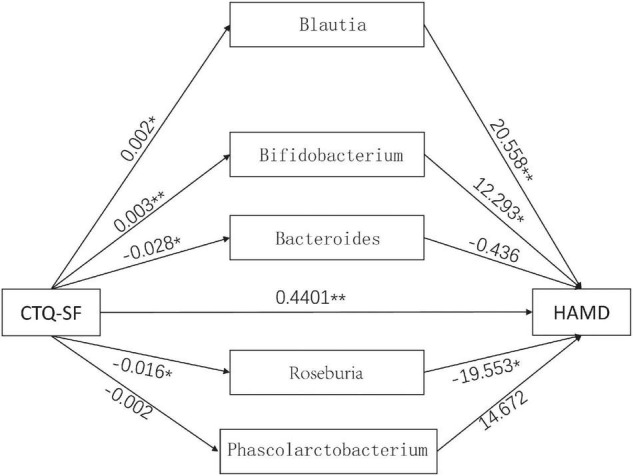
The mediation model involving the CTQ-SF score, gut microbiota and the HAMD score. The numbers in the figure represent the effect size. *P < 0.05, **P < 0.01.

To further explore the mediating effect of gut microbiota, we constructed a mediation model, with the CTQ-SF score as the independent variable, HAMD-24 scale as the dependent variable, and Blautia, Bifidobacterium, Bacteroides, Roseburia, and Phascolarctobacterium as intermediate variables (Figure 5). The results showed that the score of CTQ-SF had a significant direct effect on the score of HAMD-24 (*P* < 0.01), with the value of direct effect being 0.4401 (Table 2). The gut microbiome had a significant indirect effect, with the value of indirect effect being 0.1138. Blautia, Bifidobacterium, and Roseburia also showed significant indirect effect.

**TABLE 2:** The mediating effect of gut microbiota.

Direct effect of X on Y	Effect	SE	t	*P*	LLCI	ULCI
	0.4401	0.0609	7.2214	0.0000	0.3189	0.5613
**Indirect effect of X on Y**						
TOTAL	0.1138	0.0427	\	\	0.0424	0.2073
*Blautia*	0.0411	0.0198	\	\	0.0116	0.0952
*Bifidobacterium*	0.0432	0.0213	\	\	0.0125	0.1046
*Bacteroides*	0.0012	0.0225	\	\	−0.0427	0.049
*Roseburia*	0.0305	0.0221	\	\	0.0034	0.0898
*Phascolarctobacterium*	−0.0022	0.0179	\	\	−0.0306	0.0367

*X = CTQ-SF, Y = HAMD-24.*

## Discussion

It is well recognized that the experience of childhood maltreatment is strongly associated with depressive symptoms. However, the specific mechanism through which depression is affected by childhood maltreatment still remains understudied. Through years of in-depth study of gut microbiota, it has been found that the disturbance of gut microbiota is closely related to the pathogenesis of depression. In this study, we explored the relationship between child maltreatment, gut microbiota, and MDD. And the result showed that experience of childhood maltreatment might affect depressive symptoms through gut microbiota.

Childhood maltreatment was recognized as a risk factor for the development and maintenance of depression and was associated with increased risk of suicidal behavior (Zelazny et al., 2019). A study showed that the severity of depression was most strongly associated with EA and neglect, which were regarded as risk factors for MDD (Nelson et al., 2017). Consistent with the above results, this study found that depressive symptoms were closely related to EA and neglect, as well as PA and neglect. Different types of childhood maltreatment, such as emotional/PA and neglect, are interlinked and might be risk factors of the development of depression. Therefore, the link between different types of childhood maltreatment and depression, as well as the underlying mechanism, is worthy of further study.

According to many previous studies, patients with MDD are highly likely to have gut microbiota disorders. In this study, the α-diversity analysis showed that Simpson index and Pielou’s evenness index of HCs were significantly higher than those of MDD patients without experience of childhood maltreatment, suggesting that patients with MDD might have a lower diversity of gut microbiota than HCs. This finding was inconsistent with the results from the study by Jiang et al. (2015). By comparing the gut microbiota between patients with active MDD and HCs, they found that patients with active MDD showed higher Shannon index as compared to HCs. In another study where the shotgun metagenomics method was used to compare the gut microbiome between HCs and patients with MDD (Lai et al., 2021), the Fisher index of HCs was higher than that of MDD patients, it suggests that microbiota abundance is lower in MDD compared with HCs, while no statistical difference in Shannon index was found between the two groups. Although our results are not completely consistent with the results of the above study, our results suggested that the diversity of gut microbiota was lower in patients with MDD, as compared to HCs. We speculated that the inconsistency might be resulted from by the different sequencing methods and standards used in the studies.

The results obtained from animal models of early stress showed a significant difference in fecal microbiota between animals with and without early stress as well as affected homeostasis of gut microbiota in animals that had experienced early stress (Bailey and Coe, 1999; O’Mahony et al., 2009). In our study, there was no significant difference in α-diversity between MDD patients with child maltreatment experience, MDD patients without child maltreatment experience, and HCs. However, we found a significant difference in β-diversity between MDD patients without childhood maltreatment experience and HCs and between MDD patients with childhood maltreatment experience and HCs.

In the present study, we found that, in MDD patients without childhood maltreatment experience, the abundance of the *Actinobacteria* and *Enterobacteriaceae* was higher than that in HCs. This is consistent with the findings of Jiang et al. (2015), Chen J. J. et al. (2018), and Chen Z. et al. (2018). We also found that in the group of MDD patients with childhood maltreatment experience, the abundance of the *Bifidobacterium* was higher than that in HCs and MDD patients without childhood maltreatment experience. Similar to the study of Rong et al. (2019) our study also found that *Bifidobacterium* accumulated in depressed patients. Although many studies discussed whether probiotics could improve stress and mood, no significant findings have been found (Romijn et al., 2017). According to the above studies and the present study, the accumulation of probiotics in the body does not necessarily mean good health, and the accumulation of *Bifidobacterium* might be associated with stress in patients with depression. Therefore, the relationship between *Bifidobacterium*, depression and childhood maltreatment needs to be further studied.

A study (Naseribafrouei et al., 2014) analyzing gut microbiome of MDD patients and HCs based on Illumina deep sequencing of amplicons of 16S rRNA gene found that *Bacteroidetes, Oscillibacter*, and *Alistipes* were significantly associated with depression. In the present study, we found that *Blautia* and *Bifidobacterium* were significantly positively correlated with depressive symptoms, while *Roseburia, Bacteroides*, and *Phascolarctobacterium* were significantly negatively correlated with depressive symptoms, indicating that our results are consistent with the findings in the previous study. In addition, we found that *Blautia* and *Bifidobacterium* were significantly positively correlated not only with depressive symptoms, but also with child maltreatment, and that *Roseburia, Bacteroides*, and *Phascolarctobacterium* were significantly negatively correlated with depressive symptoms and child maltreatment. To our knowledge, these findings in our study have not been reported before.

Many studies have demonstrated the link between intestinal homeostasis and depressive symptoms. Some studies found a causal relationship between disturbance of gut microbiota and depressive symptoms using germ-free animal models, microbiota transplantation (Zheng et al., 2016; Li et al., 2019; Settanni et al., 2021) and targeted intestinal microbiota intervention (Sun et al., 2018). There are also studies showing that disturbance of gut microbiota might affect brain function by disturbing the immune system (Cryan et al., 2019), endocrine system, vagus nerve (Foster and McVey Neufeld, 2013), metabolic function (Mayer et al., 2015; Liang et al., 2018) and other pathways in the host.

Some studies used rejection, core self-evaluation and parent-child relationship as mediators to explain the relationship between child maltreatment and depressive symptoms (Song et al., 2021; Wang et al., 2021). However, most studies failed to link child maltreatment with objective indicators and physiological processes, including gut microbiota. Therefore, in the present study, we established a mediating effect model with child maltreatment as a mediator and found that *Blautia, Bifidobacterium*, and *Roseburia* had significant indirect effect.

Several studies have shown that *Blautia, Bifidobacterium*, and *Roseburia* have potential probiotic properties (Tamanai-Shacoori et al., 2017; Liu et al., 2021; Tian et al., 2022). However, on the one hand, the composition and changes of gut microbiota are related to age of host (Odamaki et al., 2016), geographic location (Nakayama et al., 2015), diet, genotype, health status, disease status, and other physiological status of the host (Mao et al., 2018); on the other hand, our study focused on the genus level, which allowed for the analyses of more species in each genus. In future works, more attention can be given to the physiological effects of gut microbiota at the species level.

## Limitation

Despite the findings and strengths of our study, some limitations should be mentioned. First, the sample size of this study is small, which might have affected the accuracy of the results. Secondly, the data of childhood maltreatment were obtained through patient inquiry, meaning that the subjects needed to recall their memories before the age of 16, which might have introduced memory bias. This, to some extent, could be addressed by recording the subtypes of child maltreatment for patients with MDD. Moreover, we used the 16S RNA sequencing method to assess gut microbiota of participants, which might not be able reach the accuracy level for those species. Finally, certain environmental factors that are difficult to control, such as diet, might have affected the gut microbiota. In future works, inclusion and exclusion criteria can be more strictly controlled to rule out the interference of environmental factors.

## Conclusion

The present study suggested that the gut microbiota of MDD patients with childhood maltreatment experience is different from that of HCs, and that the effect of childhood maltreatment on depressive symptoms might be exerted through gut microbiota and other pathways, such as genetics, immune, endocrine, etc., which can be investigated in further studies.

## Data Availability Statement

Publicly available datasets were analyzed in this study. This data can be found on NCBI with accession number: PRJNA830325.

## Ethics Statement

The studies involving human participants were reviewed and approved by the Ethics Committee of the First Hospital of Shanxi Medical University. The patients/participants provided their written informed consent to participate in this study.

## Author Contributions

NS and YuZ designed the experiments. NS, RZ, PL, MG, JY, and CY participated in the collection of clinical data and stool samples of all subjects. JW and YaZ processed the stool samples. YaZ analyzed the data and wrote the manuscript. All authors contributed to the clinical data collection and assessment.

## Conflict of Interest

The authors declare that the research was conducted in the absence of any commercial or financial relationships that could be construed as a potential conflict of interest.

## Publisher’s Note

All claims expressed in this article are solely those of the authors and do not necessarily represent those of their affiliated organizations, or those of the publisher, the editors and the reviewers. Any product that may be evaluated in this article, or claim that may be made by its manufacturer, is not guaranteed or endorsed by the publisher.

## Footnotes

1
https://www.who.int/news-room/fact-sheets/detail/depression

2
http://ghdx.healthdata.org/gbd-results-tool?params=gbd-api-2019-permalink/d780dffbe8a381b25e1416884959e88b
